# Artificial Intelligence in Intravascular Imaging for Percutaneous Coronary Interventions: A New Era of Precision

**DOI:** 10.1016/j.jscai.2024.102506

**Published:** 2025-03-18

**Authors:** Doosup Shin, Zainab Sami, Matthew Cannata, Yasemin Ciftcikal, Emma Caron, Susan V. Thomas, Craig R. Porter, Anna Tsioulias, Misha Gujja, Koshiro Sakai, Jeffrey W. Moses, Fernando A. Sosa, Richard Shlofmitz, Allen Jeremias, Ziad A. Ali, Evan Shlofmitz

**Affiliations:** a Department of Cardiology, St Francis Hospital and Heart Center, Roslyn, New York; b College of Osteopathic Medicine, New York Institute of Technology, Old Westbury, New York; c Division of Cardiology, Department of Medicine, NewYork-Presbyterian Hospital/Columbia University Irving Medical Center, New York, New York

**Keywords:** artificial intelligence, intravascular imaging, intravascular ultrasound, optical coherence tomography

## Abstract

Intravascular imaging (IVI), including intravascular ultrasound and optical coherence tomography, play a crucial role in guiding percutaneous coronary intervention by providing detailed visualization of coronary anatomy and plaque morphology. Despite substantial evidence supporting IVI use, its adoption in clinical practice remains limited for multiple reasons including limited operator experience and a lack of confidence in image interpretation. The emergence of artificial intelligence presents a promising solution to these challenges by enhancing procedural efficiency and precision, thereby potentially increasing both IVI adoption and procedural optimization. This manuscript discusses the current applications, challenges, and future directions of artificial intelligence in IVI for percutaneous coronary intervention.

## Introduction

Although percutaneous coronary intervention (PCI) is most commonly guided by angiography, this technique has inherent limitations with respect to guiding and optimizing stent implantation. Angiography provides only a 2-dimensional view of the lumen of coronary arteries, which can overlook critical details of coronary atherosclerosis. In contrast, intravascular imaging (IVI), such as intravascular ultrasound (IVUS) and optical coherence tomography (OCT), offers a more comprehensive evaluation of these morphological features, providing essential insights that angiography cannot. Growing evidence from multiple randomized trials^1^, ^2^, ^3^, ^4^, ^5^, ^6^, ^7^ supports the use of IVI during PCI, highlighting its advantages over angiography-guided PCI. A recent meta-analysis of 22 clinical trials demonstrated that IVI-guided PCI leads to significant clinical benefits, including 29% relative risk reduction in target lesion failure, 45% reduction in cardiac death, and 18% reduction in target vessel myocardial infarction compared with angiography-guided PCI.^8^ While the 2021 American College of Cardiology/American Heart Association/Society for Cardiovascular Angiography and Interventions guidelines recommend IVI as Class IIa indication,^9^ the most updated European Society of Cardiology guidelines call for routine IVI use as a Class Ia recommendation for PCI of complex lesions including left main, bifurcation, and long lesions.^10^

Despite this robust evidence, IVI remains underutilized in the United States, with an adoption rate of <15% for all PCI procedures.^11^ Several factors contribute to the low adoption rate of IVI in clinical practice, including limited operator experience, insufficient confidence in interpreting images, and the significant time investment required for accurate analysis.^11^^,^^12^ Furthermore, our previous survey revealed that only 7% of interventional fellows felt fully independent in performing and interpreting all IVI and physiology modalities, indicating that their training may not sufficiently prepare them for independent practice in these areas.^13^

The application of artificial intelligence (AI) technology in IVI has the potential to address these barriers. AI can not only assist with precise image interpretation but can also reduce the time and effort required to analyze images and obtain parameters that are crucial in pre-PCI planning and post-PCI optimization. In this review, we discuss the current applications, challenges, and future directions of AI in intravascular imaging for PCI.

## AI in IVI

AI has the potential to transform the use of IVI in clinical practice by significantly improving both its precision and efficiency. A key aspect of AI includes machine learning (ML), a set of techniques that enables AI systems to learn and adapt over time. In particular, deep learning (DL), modeled after the complex neural networks of the human brain, is increasingly being applied in cardiovascular imaging, driving advancements in automation and precision.^14^ DL algorithms can extract regular patterns from data through iterative training and apply these patterns to make predictions on new data.^15^ Most of these algorithms have been trained using expert analysis and annotations as the reference standard.^15^^,^^16^ Particularly, convolutional neural network (CNNs), a specialized DL architecture, are widely employed in various computer vision applications due to their exceptional performance in image processing, including AI-assisted IVI platforms for lesion detection and segmentation. The key advantage of CNNs over traditional pattern recognition methods lies in their deeper architecture, which incorporates numerous layers and adjustable parameters.^16^ This enables CNNs to learn features at different levels of detail and combine them to form a more comprehensive understanding through multiple layers of abstraction.^17^ Furthermore, CNNs are designed to automatically and adaptively learn the spatial hierarchies of features via backpropagation.^18^^,^^19^ With these techniques, AI can be trained to process and analyze vast amounts of imaging data with unparalleled speed and accuracy.^20^ In clinical practice, AI-driven IVI systems have the potential to provide real-time, automated interpretation of IVI, offering standardized assessments that reduce variability and enhance efficiency. For instance, AI can aid vessel segmentation, instantaneously providing the size of the vessel that can be used in selection of device size. Table 1 demonstrates examples of available IVI systems that integrate several AI-assisted features.

**Table 1:** Examples of available intravascular imaging tools using AI.

Imaging system	Modality	AI-assisted features	Potential clinical implications
AVVIGO+, Boston Scientific	IVUS	Lumen and vessel segmentation Length measurement Plaque burden detection	Selection of stent and balloon size Selection of stent length Decision for PCI, landing zone selection Post-PCI optimization
Ultreon 2.0, Abbott Vascular	OCT	EEL detection Calcium detection (arc, thickness, length)	Selection of stent and balloon size Decision for advanced calcium modification
Gentuity HF-OCT, Nipro	HF-OCT	Lumen segmentation and diameter MLA detection MSA detection, stent expansion Identification of guiding catheter	Selection of stent and balloon size Decision for PCI Post-PCI optimization Automatically removing unnecessary images and improving efficiency
HyperVue, SpectraWAVE	NIRS-OCT	Lumen and EEL detection and segmentation Plaque burden detection Quantitative lipid core detection Calcium detection (arc, thickness, length) Stent detection, MSA and expansion	Selection of stent and balloon size Decision for PCI Detection of high-risk plaque Decision for advanced calcium modification Post-PCI optimization

AI, artificial intelligence; EEL, external elastic lamina; HF, high-frequency; IVUS, intravascular ultrasound; MLA, minimal lumen area; MSA, minimal stent area; NIRS, near-infrared spectroscopy; OCT, optical coherence tomography; PCI, percutaneous coronary intervention.

## AVVIGO+ IVUS system

**Figure 1:**
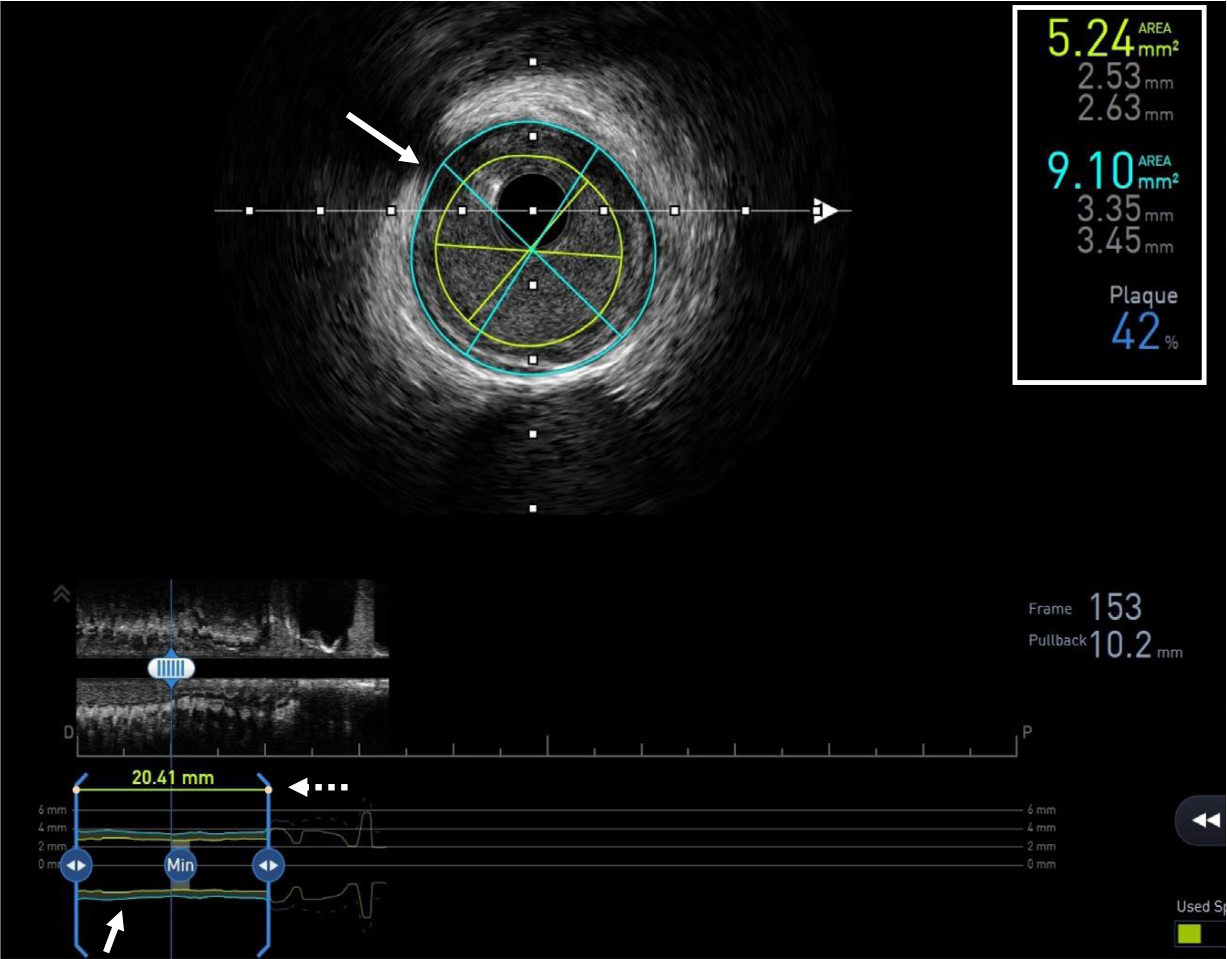
**AI-assisted features of the AVVIGO+ IVUS system.** The AI-assisted vessel segmentation (cyan blue) and lumen segmentation (yellow-green) of the AVVIGO+ IVUS System (Boston Scientific) are illustrated in the cross-sectional view (upper panel) and the longitudinal view (lower panel, white arrows). Automatic measurements of diameter, area, and plaque burden are displayed in the upper right corner (white box), assisting operators in determining the need for stent implantation, identifying landing zones, and selecting the appropriate device size. A distinctive feature of the AI-assisted AVVIGO+ system is its ability to automatically select and display lesion length, proximal and distal reference segments, and the location of the minimal lumen area in the longitudinal view (dashed white arrow), thereby aiding and streamlining percutaneous coronary intervention planning. AI, artificial intelligence; IVUS, intravascular ultrasound.

The AVVIGO+ Multi-Modality Guidance System (Boston Scientific) is an advanced platform that integrates high-definition IVUS (OptiCross) and comprehensive physiological assessments. One of the key AI-driven features of the AVVIGO+ IVUS system is automated lesion assessment (ALA), which employs ML to detect lumen and vessel borders automatically, providing precise vessel measurements (Figure 1). The ML segmentation algorithm within ALA was trained using expert analysis as the gold standard, specifically utilizing “U-Net” CNNs.^16^ In a study by Matsumura et al,^16^ ML model segmentation of lumen and vessel areas correlated well with those obtained by manually labeled segmentation performed by experts. Consequently, the system achieved a 92.4% agreement rate in selecting the appropriate balloon size when considering both lumen and vessel diameters.^16^ These findings suggest that the ALA function can significantly enhance clinical practice by reliably assisting operators to select the correct device size quickly and accurately, without the need for manual segmentation. Additionally, the system automatically calculates key parameters such as plaque burden and identifies the minimal lumen area (MLA) (Figure 1), which is crucial for pre-PCI decision-making and post-PCI optimization. For example, operators can be guided in selecting the appropriate landing zone by reviewing the automatically calculated plaque burden, as placing a stent in an area with a plaque burden >50% is associated with an increased risk of restenosis.^21^ The software highlights the minimum length needed to treat the MLA with a stent and have the adjacent proximal and distal reference segments in ≤50% plaque burden. This not only aids the novice user in selecting an optimal landing zone but also improves the workflow for the experienced user. Additionally, these features are helpful for ensuring sufficient minimal stent area (MSA) or stent expansion, which are key predictors of stent failure.^21^ Identification of the location and measurements of the MLA and MSA is a significant advance in IVUS software for the first time, providing these data objectively as opposed to depending on a user’s subjective localization of this cross-section.

## Ultreon 2.0 OCT imaging system

**Figure 2:**
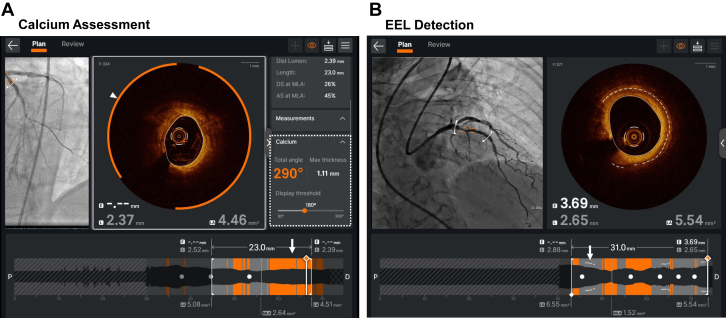
**AI-assisted features of the Ultreon 2.0 OCT system.** (A) The Ultreon 2.0 OCT system (Abbott Vascular) automatically detects calcified plaques, which are highlighted in orange in the cross-sectional image (white arrowhead), with measurements displayed in the right panel (dashed white box). Calcified plaques exceeding the operator-defined threshold (180° in this case) are marked in the angiography coregistration in the upper left panel and the longitudinal view at the bottom (white arrow). (B) AI-assisted automatic detection of the EEL is shown, represented by dashed lines in the cross-sectional image in the upper left panel. The system calculates lumen and EEL diameters to assist in selecting the appropriate device size. When the EEL is visualized over 180°, it is also shown as a white line in the longitudinal view, assisting operators in selecting relatively healthy landing zones. AI, artificial intelligence; EEL, external elastic lamina; OCT, optical coherence tomography.

The Ultreon 2.0 software (Abbott Vascular) is an AI-powered OCT imaging platform that builds upon the capabilities of its predecessor, AptiVue, by incorporating advanced AI technology. One of the unique features of Ultreon 2.0 is its ability to automatically detect calcified plaque and provide measurements, including the total calcium arc and maximum thickness (Figure 2A). Calcified plaque that exceeds the operator-defined threshold is highlighted in orange, improving viewing efficiency, and is coregistered to the angiogram with Dynamic Angio. In the longitudinal view, operators can easily evaluate the distribution of calcified plaques and accurately measure their length. These AI-assisted calcium measurements, including total calcium arc, maximum thickness, and length, are crucial in clinical decision-making, as they can be used to rapidly calculate an OCT-based calcium score to identify lesions that may benefit from advanced calcium modification therapy prior to stent implantation.^22^ Another advanced feature of Ultreon 2.0 is its capability to identify the external elastic lamina (EEL), shown as a dotted white line on both the cross-section and longitudinal profile (Figure 2B). This automated feature not only reduces variability in EEL identification^2^ but also streamlines the PCI workflow by assisting (1) determination of optimal landing zones with minimal plaque burden and greatest visibility of the EEL and (2) selection of the appropriate device size.^23^ Mean EEL measurements are automatically calculated and annotated. These AI features build upon the automated MLA, MSA, expansion, and malapposition detection, which was available in prior software versions.

A recent study has shown that these AI-assisted features of Ultreon 2.0 can enhance image interpretation process and improve viewing efficiency for both inexperienced and experienced operators.^24^ Combined with traditional algorithm-based features such as lumen detection and the identification of malapposition and stent underexpansion, these AI-driven capabilities have the potential to enhance the precision and efficiency of PCI, reduce barriers to IVI adoption in clinical practice, and promote the routine use of algorithmic approaches, such as the “MLD-MAX” strategy for IVI-guided PCI.^25^

## Gentuity HF-OCT imaging system

**Figure 3:**
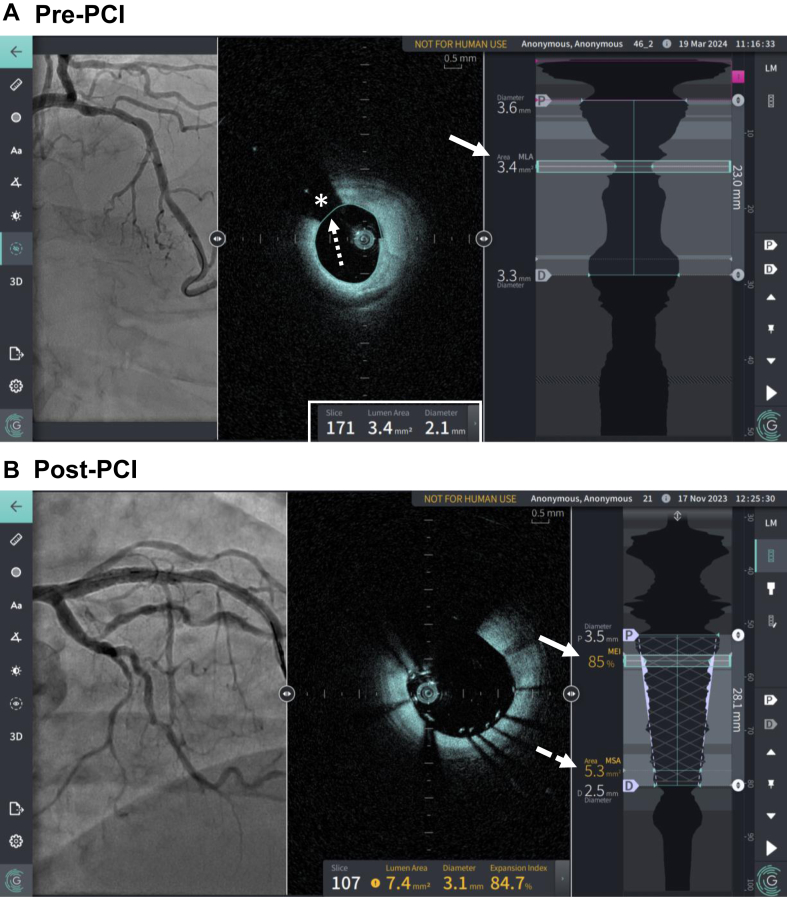
**AI-assisted features of the Gentuity high-frequency optical coherence tomography system.** (A) AI-assisted lumen segmentation provides lumen area and diameter measurements (center bottom, white box) and identifies the minimal lumen area along with its location in the longitudinal view (right panel, white arrow). Notably, the software can demarcate the lumen (white dashed arrow) even with signal attenuation caused by the presence of the guide wire (white asterisk). (B) After stent implantation, the software automatically detects the stent, calculating the minimal expansion index (white arrow) and minimal stent area (white dashed arrow), along with their locations in the longitudinal view. This aids operators in assessing and optimizing stent implantation. AI, artificial intelligence; HF-OCT, high-frequency optical coherence tomography; PCI, percutaneous coronary intervention.

The Gentuity high-frequency OCT (HF-OCT; Nipro) is a novel, fast-pullback OCT imaging system that utilizes a small Vis-Rx microimaging catheter.^26^ With its compact size (1.8F), the catheter offers enhanced crossability, while the rapid pullback feature enables a reduction in contrast volume without compromising imaging quality compared with conventional OCT systems.^27^ In addition to these benefits, the Gentuity HF-OCT system utilizes several AI-based features to assist imaging interpretation and the decision-making process (Figure 3). In the Gentuity HF-OCT system, AI is employed for high-quality lumen segmentation, even in challenging scenarios such as incomplete blood clearance, side branches, and occlusive lesions. This enables the fully automated generation of lumen profile views, allowing for the automatic identification of lumen areas and diameters in each frame, as well as determining stenosis severity and the location of the minimum lumen area throughout the pullback (Figure 3A). AI is also utilized for the automated identification of implanted stents, calculating stent expansion based on reference frames and automatically providing the minimum expansion index and MSA in real time (Figure 3B). Another useful application of AI is the automated identification of the guide catheter location. For example, in a 100 mm pullback with 40 mm inside the guide, the software automatically displays the lumen profile for the 60 mm of pullback within the artery, cropping out the 40 mm within the guide to enhance efficiency.

## SpectraWAVE HyperVue Imaging System

**Figure 4:**
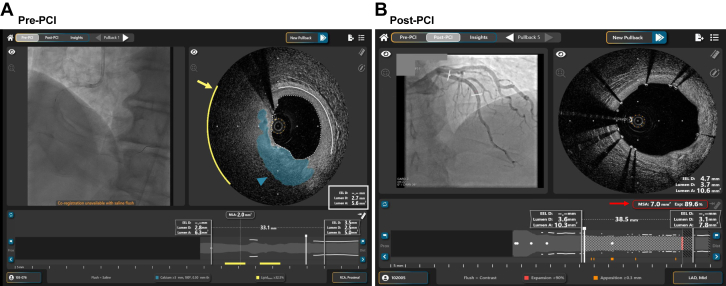
**AI-assisted features of SpectraWAVE HyperVue Imaging System.** (A) Pre-PCI optical coherence tomography imaging, obtained using a saline flush, demonstrates AI-assisted segmentations of the lumen (dashed white line) and vessel (solid white line), with corresponding measurements in the cross-sectional view. Notably, the EEL-based diameter is not calculated due to the poor visualization of the EEL, attributed to the presence of a calcified plaque, which is automatically detected and delineated in blue-green (blue-green arrowhead). Additionally, the yellow arc (yellow arrow), detected by near-infrared spectroscopy, indicates the presence of a lipidic plaque underlying the calcified area. (B) Poststent implantation, the AI software automatically detects the stent struts and calculates the minimal stent area and expansion (red arrow and box), with the respective location indicated in the longitudinal view. AI, artificial intelligence; EEL, external elastic lamina; PCI, percutaneous coronary intervention.

The HyperVue Imaging System (SpectraWAVE) utilizes the Starlight multimodality imaging catheter, combining DeepOCT with near-infrared spectroscopy (NIRS) to streamline workflow through AI and DL technologies.^28^ Key AI-driven features include automated quantitative analysis, lumen and EEL detection with dimensional assessments, quantitative lipid core detection, plaque burden analysis, calcium detection with thickness, arc, and longitudinal measurements, stent detection, and MSA/expansion assessment (Table 1). By integrating OCT and NIRS, the HyperVue AI-assisted system not only provides essential measurements for pre-PCI planning and post-PCI optimization but also enables detailed evaluation of both calcium and lipid plaques with high accuracy (Figure 4). Other clinical features that leverage the core AI technology are a rapid automated triggering of pullback based on contrast or saline flush with a high-speed pullback to minimize blood swirl and hands-free angiographic coregistration. SpectraWAVE’s fast, long scan length and precise AI-based analysis of combined OCT and NIRS images provides immediate, high-quality clinical insights, reducing contrast use and streamlining workflow efficiency.

## Limitations and future directions

Although AI and ML technologies have already been incorporated into various IVI systems, there are limitations, and a considerable journey remains to fully harness their potential. First, AI-based lumen and vessel segmentation can be affected by attenuation caused by calcified or lipid plaques, suboptimal imaging conditions, or artifacts that obscure vessel visibility. While AI and ML technologies can mitigate some of these issues, standardized imaging acquisition protocols, along with dedicated efforts and time to obtain high-quality images, remain essential. With advancements in AI for detecting various artifacts and identifying the causes of suboptimal imaging quality, the future imaging systems may offer real-time feedback and guide operators through steps to optimize imaging quality for more accurate assessments. Second, current AI-assisted imaging systems lack the ability to automatically assess important qualitative features, such as detecting stent edge dissection and differentiating thrombus from artifacts, all of which are crucial in clinical settings. As the absence of these capabilities may represent a significant barrier to the wider adoption of IVI, prioritizing the development of these features is essential for advancing the next generation of AI-assisted imaging systems. Third, while AI and ML can streamline the imaging interpretation process, operators must critically evaluate the AI-generated segmentation and assessments, ensuring the data are interpreted in clinically meaningful ways.^29^ Moreover, final decision-making will depend on the operator’s judgment, taking into account various clinical and patient-related factors, and current AI-assisted imaging systems cannot fully replace the expertise needed for IVI-guided PCI. Fourth, most of the currently available AI-assisted imaging systems have been trained using expert analysis of relatively limited data sets as the reference standard. While this has allowed for the development of functional models, ongoing efforts are needed to enhance these models, not only through continuous learning from larger and more diverse data sets but also by incorporating histopathology as the gold standard.

Despite these limitations, AI undoubtedly has the potential to significantly enhance the use of IVI and improve the precision of PCI and patient outcomes. One of the greatest potentials of AI lies in its ability to comprehensively assess and integrate a wide range of factors, including patient-specific characteristics such as genetic information and comorbidities, as well as IVI-derived lesion-level features such as high-risk plaque characteristics and hemodynamic parameters including trans-stenotic pressure gradients and wall shear stress. AI could be particularly valuable in detecting high-risk characteristics of vulnerable plaques, which can be challenging even for experts in core laboratories.^30^ As evidence grows supporting the importance of recognizing these features for clinical decision-making and prognostication,^31^^,^^32^ AI has the potential to improve both the accuracy of detection and the generalizability of these assessments in clinical practice.^15^^,^^33^ Additionally, integrating AI with computational simulations and extended reality could enable more precise interventions.^34^ By leveraging these capabilities, AI could enhance risk stratification and support tailored interventions, potentially leading to improved long-term outcomes for individual patients. As AI’s role in clinical practice and interventional cardiology continues to expand, it is imperative to address the accompanying ethical and legal considerations, particularly concerning the extent of AI’s application and the attribution of responsibility.

## Conclusion

In this review, we introduced AI-assisted features of several IVUS and OCT imaging systems and how they can help operators rapidly interpret IVI images, obtain accurate measurements and optimize PCI procedures (Central Illustration). Beyond improving the precision and efficiency of PCI, AI holds significant potential for enhancing risk stratification and enabling a personalized approach by integrating a wide range of factors, including both patient-specific and lesion-specific characteristics.

**Central Illustration:**
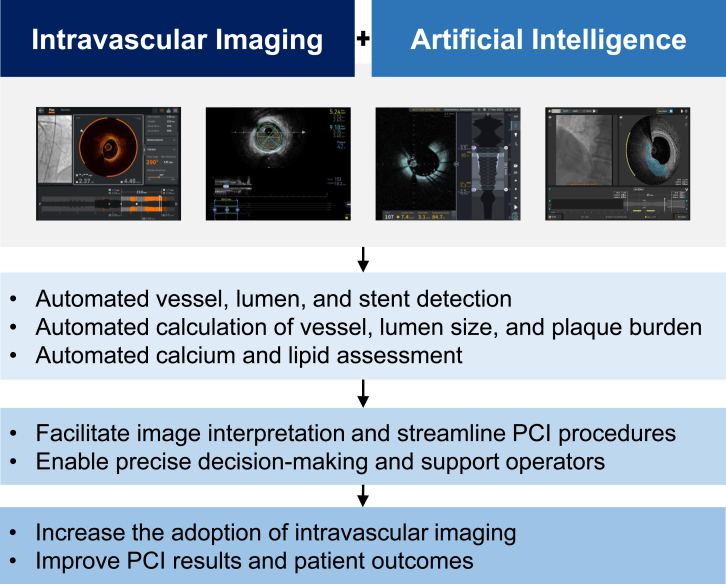
**Artificial intelligence in intravascular imaging for PCI.** Artificial intelligence is integrated into several intravascular imaging systems, enabling automated detection and quantification of vessel and lumen dimensions as well as assessment of calcium and lipid. These capabilities assist operators in image interpretation, streamline procedural workflows, and support precise decision-making during PCI. Consequently, artificial intelligence integration may not only increase the adoption of intravascular imaging but may also enhance clinical outcomes following PCI. PCI, percutaneous coronary intervention.
